# Residents' Perspectives of Pregnancy and Growing a Family During Surgical Training: A Review of the Literature

**DOI:** 10.7759/cureus.58335

**Published:** 2024-04-15

**Authors:** Isabel C Bernal, Savannah L Moon, Mayo Hotta, Martin I Newman

**Affiliations:** 1 Dr. Kiran C. Patel College of Osteopathic Medicine, Nova Southeastern University, Fort Lauderdale, USA; 2 Plastic and Reconstructive Surgery, Nicklaus Children's Hospital, Miami, USA; 3 Plastic and Reconstructive Surgery, Cleveland Clinic Florida, Weston, FL, USA; 4 Plastic and Reconstructive Surgery, Cleveland Clinic Florida, Weston, USA

**Keywords:** surgical residents, family planning, surgery, residency, pregnancy

## Abstract

As more female surgical residents choose to start families during training, concerns regarding program support and peer perceptions emerge. Delayed parenthood, stress, and even attrition can result from inadequate support systems. Database search (MEDLINE, PubMed, EMBASE) in June 2022 identified 17 relevant studies published between 2012-2022, including systematic reviews and qualitative surveys, focused on surgical residents/fellows and program directors. The thematic analysis explored themes related to supporting residents navigating parenthood. Thematic analysis of 17 studies (systematic reviews and qualitative surveys with residents/fellows and program directors) identified key recurring themes related to challenges experienced by surgical residents navigating parenthood. The themes included modified work schedules, mentorship programs, cross-coverage plans, lactation support, childcare options, and clear leave policies. By understanding these challenges and implementing tailored support strategies, surgical residency programs can foster a more inclusive and supportive environment for residents starting families. This can improve resident well-being, reduce attrition, and create a significantly more enjoyable training experience for all involved. This review aims to provide insight into residents' difficulties while pregnant or considering pregnancy and identify changes programs could implement to promote a more supportive culture for pregnant residents.

## Introduction and background

Despite historical underrepresentation, women are increasingly entering the field of surgery. While their presence in 2007 remained below 15% across specialties like general surgery, plastic surgery, and otolaryngology (ENT), remarkable progress has been achieved by 2021 [1]. The proportion of female general surgeons has nearly doubled, reaching 22.6%, and similar strides are evident in plastic surgery (17.6%), ENT (18.9%), and vascular surgery (15.4%) [2]. This encouraging trend is likely fueled by the increasing number of women entering medical school, with over half of all medical students being women in 2019 [3]. As these future physicians graduate and specialize, we can hope to see an even stronger female presence in surgery, enriching the field with diverse perspectives and talents.

However, the increasing number of women in surgery has also brought new challenges. As the gender gap in surgical residency narrows and female representation increases in a demanding field that has not traditionally attracted women, more women decide to start a family during residency and fellowship training. Supporting these residents' and their families' health and well-being has become a priority, as evidenced by the Accreditation Council for Graduate Medical Education's (ACGME) recent implementation of parental leave regulations [4]. Although this policy marks a positive step, many areas remain where additional support may be welcome for trainee parents that their training programs could facilitate.

For decades, female surgical residents have faced a significant trade-off between career advancement and starting a family. The average age of first pregnancy in these demanding specialties remains 33 years, considerably higher than the national average of 27 [5]. This delay often coincides with increased difficulty conceiving, with over 30% of female surgeons reporting fertility issues compared to the national average of 10.9% [6]. Driven by this pressure and evolving personal priorities, a growing trend sees more residents choosing to start families during training itself. A survey conducted by Turner et al. (2012) among female surgeons highlights this shift, noting a rise in pregnancies among recently trained surgeons compared to those who finished over 20 years ago [7]. Recognizing this changing landscape and the specific needs of residents navigating parenthood, this paper explores the various challenges they encounter and advocates for increased support from surgical residency programs.

Years of dedicated service and practice are required to become a surgeon, making surgical training inherently demanding and time-consuming. This, coupled with the persistent lack of female mentors and negative perceptions of work-life balance, creates a challenging environment for female medical students considering a career in surgery [8]. These systemic barriers can also deter residents from starting families during training, potentially exacerbating the workforce imbalance within the field.

The challenges faced by residents navigating parenthood range from personal decisions about timing and fertility concerns to logistical hurdles like managing training demands with prenatal care and finding childcare. Additionally, cultural factors like negative perceptions of pregnancy and potential stigma can negatively impact resident well-being. This dissatisfaction is reflected in a study where 39% of pregnant resident surgeons reported considering leaving their programs [9]. While existing research explores some of these challenges and proposes solutions such as leave policies and childcare options, they lack a comprehensive approach to address the complex well-being issues faced by female surgical residents. This paper aims to fill this gap by offering a holistic framework for supporting resident well-being and promoting a more inclusive and supportive environment for parenthood within surgical training programs [10]. By exploring the experiences of residents encountering pregnancy or family planning concerns with surgical training, the objective of this review is to provide insight into residents' difficulties and recommend holistic and effective strategies that residency programs can implement to support residents navigating parenthood.

## Review

Methods

Search Strategy and Study Selection

**Figure 1:**
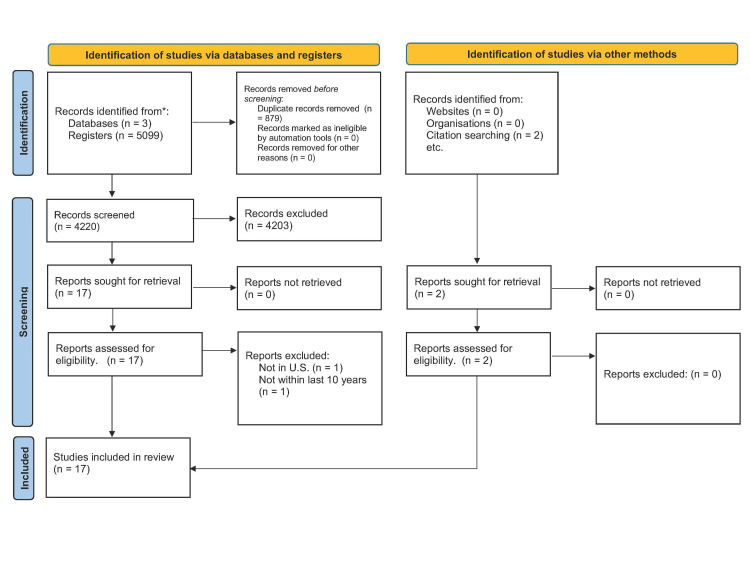
PRISMA flow chart of databases: PubMed, Medline, and EMBASE PRISMA - preferred reporting items for systemic review and meta-analyses

A narrative qualitative review was performed in June 2022 to understand further the experiences of surgical residents who grow their families in training and highlight possible solutions. To ensure transparency, the 2020 preferred reporting issues of systemic review and meta-analyses (PRIMA) guidelines were followed in this review [11]. A database search of literature published from 2012-2022 was conducted using MEDLINE, PubMed, and EMBASE. The article abstracts were filtered based on specific inclusion and exclusion criteria to refine the selection further. Then, a full-text filter was applied to the remaining articles. Finally, a PRISMA flow chart was created using the EndNote data results to visualize the article selection process (Figure 1).

Two independent reviewers evaluated the abstracts of the articles according to predefined criteria to determine eligibility for inclusion. The selected articles underwent cross-referencing, and any discrepancies in article inclusion were addressed through discussion. The inclusion criteria included the following conditions: United States (US) surgical residency programs and pregnancy or family planning during training. Any articles that did not meet these criteria were excluded from the review. Next, a full-text filter was applied to the remaining articles, considering the following criteria: inclusion of surgical and sub-surgical specialties residents, fellows, or program directors, discussion of pregnancy during training, and exploration of family planning during training. Articles that did not discuss at least one of the following aspects were excluded: hardships experienced during pregnancy, challenges faced upon returning to work, support received during pregnancy or upon returning to work, and articles published before 2012. This systematic review approach ensures a comprehensive and representative selection of relevant studies, minimizing potential bias and enhancing the generalizability of our findings.

Data Extraction and Analysis

The data was extracted by the same two reviewers from studies that met the inclusion criteria. A thematic analysis was used to identify recurring themes from the data. To do this, an in-depth analysis of the content of the articles was conducted. Survey responses were broken down to understand the nuances of the resident's experiences. The specific reporting patterns we focused on pertained to modified work schedules, mentorship, cross-coverage plans, lactation rooms, and support for nursing, childcare, and leave policies. The themes were then reviewed, and any disagreements were discussed.

Results

The search resulted in 5099 articles, of which 4220 remained after deduplication using EndNote. By applying inclusion criteria, we further narrowed this to 17 eligible studies, summarized in Table 1. These studies included both systematic reviews and qualitative surveys relevant to surgical residents/fellows and program directors. Notably, the surveys covered a broad range of surgical specialties, including general surgery, plastic surgery, orthopedic surgery, urology, neurosurgery, vascular surgery, thoracic surgery, otolaryngology, colorectal surgery, and trauma surgery.

**Table 1:** Summary of studies included in the review

Author	Study Design	Objective	Specialties	Participants
Rangel et al, 2018 [12]	Qualitative survey	Directly assess the resident experience of childbearing during training.	General surgery, trauma and acute care surgery, breast, pediatric surgery, plastic and reconstructive surgery, colorectal, vascular, bariatric and minimally invasive, cardiothoracic, surgical oncology, endocrine, transplant surgery	188 residents/fellows
Todd et al, 2020 [10]	Systematic review	Examine common themes and synthesize data surrounding pregnancy and parenthood during surgical residency training.	General surgery, orthopedic surgery, cardiothoracic surgery, otolaryngology, plastic surgery, and urology	14,114 residents and 345 program directors
Compton et al, 2021 [13]	Qualitative review	Create a comprehensive guide for both orthopedic trainees and faculty surrounding issues of pregnancy and parental leave.	Orthopedic surgery	N/A
Mulcahey et al, 2019 [14]	Qualitative review	Determine the perceptions of and experiences with pregnancy and parenthood among female orthopedic surgery trainees.	Orthopedic surgery	190 residents
Harnsberger et al, 2019 [15]	Review	Address the physical, logistical, and financial challenges facing the pregnant surgeon.	Surgical specialty not disclosed	N/A
Rangel et al, 2021 [16]	Qualitative review	Describe the incidence of infertility and pregnancy complications among female surgeons in the US and to identify workplace factors associated with increased risk compared with a sociodemographic ally similar non-surgeon population.	Surgical specialty not disclosed	151 residents/fellows (26 males, 125 females)
Gupta et al, 2020 [17]	Qualitative survey	Report survey results, evaluate the findings in the context of the growing literature on pregnancy during training and practice from other surgical specialties, and provide recommendations to advance a more equitable workforce.	Neurosurgery	52 residents (52 females)
Shifflette et al, 2018 [18]	Qualitative survey	Show the impact that pregnancy has on the female resident and the general surgical program.	General surgery	22 residents (22 females)
Kenyon et al, 2021 [19]	Qualitative survey	Assess the urology program director's perception of pregnancy during residency training via self-reported opinions, evaluate the urology program director's awareness of parental leave at their institution, and gauge the program director's interest in formal policy implementation.	Urology	63 program directors
Garza et al, 2017 [20]	Qualitative survey	Identify the challenges plastic surgery program directors face in accommodating pregnant residents and based on results, to provide guidance in developing solutions.	Plastic and reconstructive surgery	54 plastic surgery program directors
Rangel et al, 2018 [21]	Qualitative survey	Determine factors associated with professional dissatisfaction for childbearing residents.	Surgical specialty not disclosed	188 female residents/fellows
Rangel et al, 2018 [9]	Qualitative analysis	Characterize the perspectives of pregnant surgical residents in-depth and identify positive influences that improve the experience of childbearing trainees through qualitative analysis of open-ended questions.	Surgical specialty not disclosed	188 female residents/fellows
Altieri et al, 2019 [22]	Qualitative survey	Examine the perceptions of current surgery residents regarding parental leave.	General surgery, urology, orthopedics, plastic surgery, cardiothoracic, otolaryngology or ear, nose, and throat surgery, vascular, and neurosurgery	2188 residents (1049 males, 1041 females)
Castillo-Angeles et al, 2021 [23]	Qualitative survey	Describe the perspective and experience of US surgical program directors regarding maternity leave and postpartum support for surgical residents.	General surgery	40 program directors (28 males, 12 females)
Polan et al, 2022 [24]	Quantitative review	Provide current literature on parental leave, breastfeeding, and childcare in the field of obstetrics and gynecology.	Obstetrics and gynecology	N/A
Bourne et al, 2019 [25]	Qualitative survey	Provide baseline information about reproductive issues in plastic surgery trainees, specifically focusing on obstetrical complications, parental leave, breastfeeding, childcare, and infertility.	Plastic and reconstructive surgery	307 residents (167 females, 140 males)
Magudia et al, 2020 [26]	Qualitative survey	Better understand trainee attitudes, plans, and experiences relating to parenting overall and as related to gender, specialty, and other individual characteristics in greater detail than previously available.	Surgical specialty not disclosed	83 residents

The recurring themes emerged from the studies, including diverse perspectives on modified work schedules, mentorship programs, cross-coverage plans, lactation support, childcare options, and clear leave policies. Interestingly, while discussed less frequently, the management of infertility and the length of maternity and paternity leave were also highlighted as important issues. These findings are summarized below with recommendations (Table 2).

**Table 2:** Recommendations from pooled responses that mentioned or regarded mentorship programs, cross-coverage plans, lactation rooms, and support for nursing, childcare, and clear leave policies

Modified work schedule	Mentorship programs	Cross-coverage plans	Lactation rooms and support for nursing	Childcare	Clear leave policies
Residents and program directors should collaborate to modify rotation schedules, potentially including lighter workloads upon returning from parental leave to ease the work-life balance adjustment.	Implement individualized mentoring within each program. Prioritize matching pregnant residents with in-house attendings or senior residents for tailored support.	Offer alternative coverage options like staff, cross-coverage services, or advanced providers for sick or pregnant residents, easing the load on co-residents [10,30].	High-speed pumps, skins, microwaves, refrigetarors, and integrated workspaces create optimal lactation rooms, empowering mothers to seamlessly continue patient care [16].	Flexible childcare hours in facility centers, including earlier opening times or later closing options.	A transparent and standardized parental leave policy should be introduced to all during onboarding.
Third-trimester schedule modifications are crucial, as long operating hours increase major complication risks [16]. Fixed day shifts (8 am - 6 pm) should be prioritized over lengthy night shifts or unpredictable rotating schedules, which demonstrably increase the risk of preterm delivery, low birth weight, preeclampsia, and gestational hypertension [18].	Promote the American College of Surgeons (ACS) Women in Surgery Committee's mentorship program through wellness initiatives, connecting women surgeons with experienced mentors [31].	Prioritize alternative solutions to "make-up" calls for missed time, given the dangers of long hours (>40 hrs/week) and extra nights linked to higher miscarriage risks. Programs should establish flexible "missed call" management [14,16].	Schedules should allow for lactation breaks. Per the CDC, feeding occurs every two to four hours initially and pumping should mirror the infant's schedule for optimal milk production [32].	Large institutions could offer subsidized childcare for residents with inflexible schedules, adjusted to resident salaries.	Offer residents open discussions about extended training and the American Board of Surgery (ABS) flexibility options.
Incorporate formal ergonomic consultations into wellness programs to mitigate musculoskeletal injury risks. Studies show pregnant surgeons face higher injury rates compared to non-surgeons, highlighting the need for preventative measures [16].		Offering compensation for taking additional calls to co-residents might reduce the negative perception surrounding pregnant residents.	Flexible case-coverage policies incorporating pump breaks, tailored to program needs, could empower residents to maintain surgical involvement.	Offer different subsidized childcare.	

Modified Work Schedule

Reviewing studies on pregnant surgical residents, Rangel et al. found alarming higher risks of complications like preeclampsia and preterm labor compared to non-surgeon women [12]. Notably, prolonged work hours and demanding call schedules during the final trimester were pinpointed as primary culprits [10]. Despite these risks, most female residents maintain unmodified schedules [9,27]. Interestingly, of the 17 articles examined, a majority of respondents in 11 favored modifiable work schedules to improve the situation for pregnant residents. Their proposed modifications, detailed in Table 2, offer potential solutions.

Mentorship Program

The stigma surrounding pregnancy was a dominant concern in surveys. Nearly 75% of respondents witnessed negative comments about pregnant trainees [9]. Program directors revealed that most residents become pregnant during their second or third year of training [18], with over half of residents feeling pressured to plan them during nonclinical time [9]. Mentoring programs emerged as a key solution, helping residents navigate challenges like managing negative perceptions, post-leave work reintegration, and work-life balance [10]. Ideally, mentors should be surgeon mothers offering experience-based advice on balancing commitments, setting realistic expectations, and providing a safe space for discussion [16]. Eleven out of the 17 reviewed papers highlighted the importance of mentoring programs, with majorities endorsing the specific elements detailed in Table 2.

Cross-Coverage Plans

Early notification of pregnancy allows program directors to adjust rotation schedules for both pregnant residents and soon-to-be fathers. However, unexpected pregnancy-related complications can still arise, resulting in unplanned absences. To avoid burdening co-residents and perpetuating negative pregnancy stigma, programs should have contingency plans in place [10]. Cross-coverage options were favored by respondents, with key recommendations included in Table 2.

Lactation Rooms and Support for Nursing

The American Academy of Pediatrics (AAP) recommends exclusive breastfeeding for the first six months, highlighting its benefits for infant health and reduced risk of infections and chronic conditions [28]. While nearly all surveyed residents (95.6%) valued breastfeeding, limited access to lactation rooms remains a hurdle, with location and availability being a major concern [9]. Dedicated lactation spaces are crucial for maintaining resident productivity, as emphasized in 11 out of 17 reviewed articles, which are detailed in Table 2.

Childcare

Resident parents often face a tough choice: balancing long work hours with caring for a newborn, especially when far from family support [24]. Childcare emerges as a vital resource in over 60% of studies, promoting work-life balance and success in surgical training. While institutions offer support like preferential daycare enrollment, discounted rates, or backup services, a survey by Rangel et al. (2018) found these options inadequate [12]. Eighty-five percent of women felt the offered daycare hours didn't fit their demanding schedules. Surveyed residents emphasized the crucial aspects of childcare outlined in Table 2.

Clear Leave Policies

Ideal leave policies for resident parents evolve. The AAP recommends 12 weeks, while the ACGME mandates a minimum of six weeks of paid leave effective July 2022. Residents often found pre-ACGME policy leave insufficient. Respondents valued clear policies, emphasizing their role in supporting parenthood during training. Easy access to program leave policies helps residents arrange the leave with less stress, fostering better team communication and potentially encouraging earlier childbearing with fewer complications [14,15]. Notably, the American Board of Surgery updated its policy in 2021-22, offering four weeks off per year during the first three years and an additional four in the last two years of training [29]. Ninety-four percent of studies and the majority of surveyed residents favored the points outlined in Table 2. 

Management of Infertility

Surgical residents face higher infertility rates, potentially due to later first pregnancies. This delay could increase reliance on expensive and time-consuming assisted reproductive technologies (ART) like in vitro fertilization, intrauterine insemination, and the use of clomiphene [25]. Residents often require time off for procedures and tests, potentially impacting their clinical duties [16]. In states without mandatory insurance coverage for ART, institutions may consider offering financial aid or additional insurance options to alleviate this burden on residents seeking to start families.

Length of Parental Leave

While over 75% of women felt six weeks of maternity leave is insufficient, extending training through postponement can disrupt program schedules and delay new fellows. This poses a difficult trade-off for programs and residents [16]. Interestingly, despite desiring longer leave, most residents avoid it due to concerns about lower exam scores, potentially impacting career progression as demonstrated in the Shifflette et al. study [18]. Residents instead propose flexible part-time training options to minimize career disruptions associated with pregnancy [24]. This could improve the negative perception of pregnancy interrupting and delaying training.

Challenges and Solutions

The proposals for solutions, suggested by both residents and program directors, were directly extracted from the studies and subsequently analyzed. These proposed solutions, along with insights from a literature review of policy guidelines, expert opinions, and considerations of practicality and feasibility, are summarized in Table 2.

Discussion

This review identified several key challenges faced by residents navigating parenthood during surgical training. Demanding schedules often interfere with prenatal care and fertility treatments, while inflexible work arrangements and negative perceptions from colleagues create additional stressors. Additionally, residents often lack access to adequate lactation support and childcare options, further hindering their well-being. While this study focused primarily on female residents, it's crucial to acknowledge that male residents may face similar or unique challenges requiring tailored support.

Beyond acknowledging these challenges, this review aims to highlight areas where residency programs can implement changes to better support residents considering or experiencing parenthood. This includes flexible scheduling options like part-time rotations or protected time for appointments, mentorship programs to combat negative perceptions and provide guidance, and dedicated lactation rooms with resources and support. Additionally, exploring childcare subsidies or partnering with local daycare facilities can significantly alleviate challenges for resident parents. Implementing these changes can lead to numerous benefits for both residents and programs. Improved resident well-being can translate to reduced attrition rates, increased focus and productivity, and ultimately a more positive and rewarding training experience. Furthermore, fostering a family-friendly environment can attract a more diverse workforce, enriching the field with individuals from various backgrounds and experiences.

While this review provides valuable insights, further research is needed to explore specific areas in more detail. Investigating the impact of pregnancy on resident performance, for example, could inform evidence-based recommendations for workload adjustments or modified evaluations. Additionally, research specific to high-risk pregnancies in this population would be crucial for developing appropriate risk management strategies.

By implementing the recommended changes and actively participating in future research, graduate medical education and individual surgical programs can create a more supportive and inclusive environment for all residents, fostering not only successful careers but also fulfilling personal lives.

Limitations

This review has several limitations to consider. Firstly, the generalizability of findings is somewhat limited due to the variation in the representation of surgical subspecialties. While the overall sample size was substantial (over 5,000 residents and fellows), a significant portion of studies lacked specific subspecialty data and were classified as "unspecified". This makes it difficult to draw definitive conclusions for individual subspecialties. Additionally, the review aimed to identify the experiences of both male and female residents. However, only a few of the studies included data from male residents. This is important as they may face different challenges and require tailored support systems. Future research should specifically include male residents to gain a more comprehensive understanding of their experiences.

Secondly, the reliance on subjective data from surveys introduces potential biases, such as recall bias and social desirability bias. Respondents might provide answers they perceive as more socially acceptable, affecting the accuracy and reliability of the findings. While qualitative analysis methods aim to mitigate subjectivity, limitations inherent in this approach should be acknowledged.

Despite these limitations, this review provides valuable insights into the challenges faced by residents considering or experiencing parenthood. Addressing these limitations in future research will further enhance the understanding of this important topic and inform the development of effective support systems for residents navigating parenthood within surgical training.

## Conclusions

This review explores the challenges surgical residents face when starting families during training. It highlights the need for changes to existing policies to support healthy pregnancies and acknowledges the personal and professional difficulties such residents encounter. Understanding these barriers can equip programs to improve resident well-being, reduce attrition, and enhance the overall experience for trainees seeking parenthood. The analysis reveals a complex web of personal and professional hurdles intertwined with work-life balance, healthcare policies, and the specific demands of surgical specialties. These factors significantly impact the well-being and career of residents choosing to start families during this demanding period.
